# Data-driven classification of patients with primary progressive aphasia

**DOI:** 10.1016/j.bandl.2017.08.001

**Published:** 2017-11

**Authors:** Paul Hoffman, Seyed Ahmad Sajjadi, Karalyn Patterson, Peter J. Nestor

**Affiliations:** a Centre for Cognitive Ageing and Cognitive Epidemiology (CCACE) & Department of Psychology, University of Edinburgh, UK; b Department of Neurology, University of California, Irvine, Irvine, USA; c Department of Clinical Neurosciences, University of Cambridge & MRC Cognition & Brain Sciences Unit, Cambridge, UK; d German Center for Neurodegenerative Diseases (DZNE), Magdeburg, Germany

**Keywords:** Primary progressive aphasia, Semantic dementia, Non-fluent aphasia, Logopenic aphasia, Frontotemporal dementia, Alzheimer’s disease

## Abstract

Current diagnostic criteria classify primary progressive aphasia into three variants–semantic (sv), nonfluent (nfv) and logopenic (lv) PPA–though the adequacy of this scheme is debated. This study took a data-driven approach, applying k-means clustering to data from 43 PPA patients. The algorithm grouped patients based on similarities in language, semantic and non-linguistic cognitive scores. The optimum solution consisted of three groups. One group, almost exclusively those diagnosed as svPPA, displayed a selective semantic impairment. A second cluster, with impairments to speech production, repetition and syntactic processing, contained a majority of patients with nfvPPA but also some lvPPA patients. The final group exhibited more severe deficits to speech, repetition and syntax as well as semantic and other cognitive deficits. These results suggest that, amongst cases of non-semantic PPA, differentiation mainly reflects overall degree of language/cognitive impairment. The observed patterns were scarcely affected by inclusion/exclusion of non-linguistic cognitive scores.

## Highlights

• There is current controversy over how to classify PPA variants.
• We used a k-means clustering algorithm, blind to diagnosis, to divide patients.
• Patients grouped based on similarities in linguistic and neuropsychological profile.
• One cluster of patients with selective semantic impairment.
• Two clusters with non-semantic profile, differentiated by overall level of language/cognitive impairment.

## 1 Introduction

Primary progressive aphasia (PPA) is an umbrella term which refers to a range of patients with neurodegenerative disease in whom language impairments are the most salient and clinically significant feature (Gorno-Tempini et al., 2011, Mesulam, 2001). This broad diagnostic class encompasses individuals in whom language impairments, clinical needs and underlying pathology are all diverse, and thus efforts have been made to sub-divide them into more homogeneous groups. Historically, two distinct PPA syndromes were recognised. In semantic variant PPA (svPPA, often termed semantic dementia), speech remains fluent and largely intact in both phonological and grammatical structure until late in progression; but loss of semantic knowledge results in prominent difficulties in both language comprehension and production (Hodges and Patterson, 2007, Hodges et al., 1992, Snowden et al., 1989). Conversely, the defining symptoms of non-fluent/agrammatic variant PPA (nfvPPA) are effortful speech production, speech sound errors and agrammatism (Grossman et al., 1996, Knibb et al., 2006, Ogar et al., 2007). Single-word comprehension typically remains intact in nfvPPA for a considerable time. Both syndromes have been linked with frontotemporal lobar degeneration (FTLD) pathology (Gorno-Tempini et al., 2011).

It has also been known for some time that a substantial proportion of PPA patients fail to show the typical features of either svPPA or nfvPPA, despite presenting with language deficits as the leading clinical symptom. Alzheimer disease (AD) pathology is more common among these individuals (Leyton et al., 2011). These findings led Gorno-Tempini et al. (2004) to propose a third variant – logopenic PPA (lvPPA) – characterised by poor sentence repetition and a loss of fluency that has been attributed to poor verbal working memory rather than the motor speech deficits observed in nfvPPA (Gorno-Tempini et al., 2008).

This tripartite division of PPA patients was codified in a set of diagnostic recommendations that set out inclusion and exclusion criteria for each variant (Gorno-Tempini et al., 2011). Doubts have been raised, however, regarding the adequacy of these criteria to capture the full diversity of impairments in PPA. In a recent prospective study of 46 PPA patients, Sajjadi, Patterson, Arnold, Watson, and Nestor (2012) reported that rigorous application of the proposed diagnostic criteria identified only two patients whose linguistic profile was consistent with lvPPA. Furthermore, 41% of patients could not be classified at all, either because they did not meet the requirements for any of the variants or because they qualified for more than one. Studies from other centres have identified somewhat higher proportions of lvPPA patients among their samples but have also found substantial numbers of unclassifiable patients (16% in Gil-Navarro et al., 2013; 17% in Harris et al., 2013; 20% in Mesulam, Wieneke, Thompson, Rogalski, & Weintraub, 2012; 31% in Wicklund et al., 2014). In response to these findings, some authors have proposed a fourth “mixed PPA” class for patients who cannot otherwise be classified, usually because they exhibit a combination of semantic and grammatical impairments (Mesulam and Weintraub, 2014, Sajjadi et al., 2012). In a follow-up investigation by Sajjadi, Patterson, and Nestor (2014), the 14 mixed PPA patients were shown to have a left temporoparietal distribution of atrophy that closely resembled that previously reported for lvPPA. The authors suggested that AD was the most likely underlying pathology in these cases, but that the linguistic profile of Azheimer-related aphasia is more diverse than that prescribed by the confines of the lvPPA diagnosis.

In the present study, we applied a novel analysis approach to the PPA cohort previously reported by Sajjadi et al. (2012). As discussed earlier, Sajjadi et al. investigated presentations of PPA through rigorous application of the currently accepted diagnostic criteria. Here, we approached the issue of PPA classification from a rather different, data-driven perspective. We applied statistical data-clustering methods that disregarded specific diagnostic criteria and instead grouped patients together if they showed a similar pattern of spared and impaired language and neuropsychological features. This allowed us to ask (a) how many distinct forms of PPA can be identified by a data-analytic technique that is blind to clinical diagnosis and (b) how well do these forms compare with the conventional diagnostic categories currently in use.

While some previous studies have used data-clustering approaches to investigate structure within PPA (Knibb et al., 2006, Leyton et al., 2014, Machulda et al., 2013, Wicklund et al., 2014), the present study extends this approach in at least three important ways. First, unlike previous studies we used k-means clustering rather than hierarchical cluster analysis to group patients. Hierarchical cluster analysis works by grouping and separating patients at a number of different levels simultaneously. This provides a useful visual guide to the relationships between patients but with the limitation that it is difficult to determine which level of the hierarchy offers the most parsimonious account of the data. In contrast, the k-means technique partitions the cohort into a fixed number of clusters, with the number of clusters controlled by the researcher. The explanatory power of the clustering solution (in terms of percentage of variance explained) can be compared across solutions with different numbers of clusters, allowing the researcher to determine how many clusters are required to provide the most parsimonious account of the data (Jain, 2010). By using this technique, we were able to ask whether the tripartite system advocated by the consensus criteria was supported by the patterns of spared and impaired function in our PPA cohort.

The second advance is that we applied cluster analytic techniques to a large and heterogeneous sample of 43 PPA patients, including those with all of the three proposed variants and those with mixed PPA. This allowed us to assess the existence of coherent symptom groupings across the entire spectrum of PPA. In contrast, previous data-driven analyses have either focused only on lvPPA (Machulda et al., 2013), have excluded svPPA (Leyton et al., 2014) or have only considered unclassifiable patients (Wicklund et al., 2014).

Finally, we considered a wider range of linguistic, cognitive and speech production measures than were included in earlier data-clustering studies or in previous analyses by Sajjadi et al. (2012). In addition to performance on neuropsychological tests of language abilities, we included quantitative measures of connected speech. Speech production is an important part of the clinical picture in PPA and a valuable diagnostic tool, with characteristic changes in speech quality associated with each variant (Ash et al., 2013, Sajjadi et al., 2012b, Wilson et al., 2010). We also included tests of non-linguistic cognitive abilities. These do not feature in the current consensus criteria but a number of authors have noted that general cognitive deficits are more common in lvPPA or Alzheimer-related PPA, relative to the other variants (Leyton et al., 2013, Teichmann et al., 2013). Other studies have reported that non-verbal test scores do not discriminate between pathologically-confirmed cases of FTD and AD (Xiong et al., 2011). Thus, the potential diagnostic value of considering a patient’s extra-linguistic neuropsychological profile remains an open question. By comparing clustering results that included or excluded non-linguistic test scores, we were able to assess whether these measures improved the ability of the clustering algorithm to discriminate distinct forms of PPA.

## 2 Method

### 2.1 Participants

Our participants comprised 43 patients with a clinical diagnosis of PPA, prospectively recruited over a two-year period (2009–2011) from memory clinics held at Addenbrooke’s Hospital, University of Cambridge, UK. All patients met the basic criteria for PPA. Non-degenerative pathologies were excluded using MRI, except in three patients who had CT because MRI was contraindicated. These patients were first reported by Sajjadi et al. (2012), who classified them through strict application of the Gorno-Tempini et al. (2011) criteria, by which 14 patients were diagnosed with svPPA, 12 with nvfPPA and 2 with lvPPA. The remaining 15 patients could not be classified, either because they did not meet criteria for any of the proposed variants or because they fitted the criteria for more than one. We refer to these patients as mixed PPA.

In addition, 30 healthy controls were recruited, matched to the patient group for age and educational level. All were free of cognitive symptoms and neurological or psychiatric illnesses and performed normally on the Addenbrooke’s Cognitive Examination – Revised (Mioshi, Dawson, Mitchell, Arnold, & Hodges, 2006).

### 2.2 Standard protocol approvals, registrations, and patient consents

Written informed consent was obtained from the participants and, where appropriate, their next of kin. The study was approved by the Cambridge regional ethics committee.

### 2.3 Neuropsychological and language assessments

Patients and controls completed a detailed neuropsychological battery described by Sajjadi et al. (2012; see Supplementary Tables 1 for full details). This was focused mainly on aspects of linguistic processing impaired in different forms of PPA: repetition and verbal short-term memory, syntax, verbal and non-verbal semantic knowledge and lexical retrieval. In addition, some tests of general cognitive function, visuospatial ability and episodic memory were included. These particular cognitive domains were targeted because it has been suggested that a continuum exists between lvPPA, posterior cortical atrophy and typical AD (Crutch et al., 2013, Migliaccio et al., 2009). It was therefore possible that impairments to visuospatial function and/or episodic memory would be instrumental in distinguishing lvPPA patients from other PPA variants.

In addition, samples of connected speech were recorded from each participant during a picture description task and a semi-structured interview. These were analysed for their linguistic content as described elsewhere (Sajjadi et al., 2012a, Sajjadi et al., 2012b; see Supplementary Table 2 for details).

### 2.4 Statistical analyses

Data entering our analyses comprised scores on the neuropsychological tests and speech markers obtained through analysis of connected speech samples. Prior to analysis, error rates from the speech samples were arcsin-transformed to reduce skew. Where necessary (i.e., in the case of error rates and reaction times) scores were reversed so that higher values always signified better performance.

As a preliminary step, test scores and language markers for all 43 patients were subjected to a principal components analysis (PCA) with varimax rotation (performed in SPSS version 20). Our battery comprised a wide range of individual tests and measures, many of which probed overlapping linguistic abilities. The goal of the PCA was to reduce the complexity of this dataset, by aggregating the measures into a smaller number of underlying cognitive/linguistic factors. The outcome of this analysis was used to compute a factor score for each patient in each linguistic/cognitive domain. All speech markers and neuropsychological test scores were entered into the PCA with the exception of scores on the Mini-Mental State Examination (MMSE; Folstein, Folstein, & McHugh, 1975) and Addenbrookes Cognitive Examination – Revised (ACE-R; Mioshi et al., 2006), since these general assessments span a range of cognitive domains. We used the results of the PCA to aid interpretation of the cluster analyses, described next, which form the basis of the present study.

K-means clustering was used to divide the patients empirically into distinct groups, based on similarity in their neuropsychological/linguistic profiles. Data from the 43 patients (including all speech markers and neuropsychological test scores but again excluding MMSE and ACE-R scores) were entered into k-means analyses using R. For any given *k*, the clustering algorithm partitions the patients into *k* clusters in such a way as to maximise the similarity of patients within each cluster and minimise the similarities between clusters. We repeated this computation several times, varying *k* between two and ten. Our next challenge was to decide which of these solutions provided the best account of the data – i.e., how many clusters most effectively partition the patients into coherent groups. Our primary method of determining this was visual inspection of the increase in variance explained through the addition of each new cluster and identification of the elbow in this graph, i.e., the point beyond which the addition of further clusters explains little additional variance (Milligan & Cooper, 1985). As an additional check, we also employed a model-based clustering method that determined the optimum number of clusters by maximising the Bayesian Information Criterion (Fraley & Raftery, 2007). Both methods suggested that there were three distinct clusters. To investigate the characteristics of these clusters of patients, we (a) compared the clinical diagnoses of patients assigned to each cluster, (b) analysed their cognitive and linguistic profiles by plotting their mean factor scores from the earlier PCA and (c) compared their scores on the MMSE and ACE-R (which were not included in the k-means computations).

Finally, we repeated the k-means analyses but this time excluded scores from six non-linguistic neuropsychological tests (cube analysis from the VOSP (Warrington & James, 1991), copy and recall of the Rey complex figure, address recognition on the ACE-R, the Trails A test and CANTAB paired associate learning (Robbins et al., 1994)). As discussed in the Introduction, extra-linguistic cognitive abilities do not form part of the current diagnostic criteria for PPA but some investigators have suggested that they are useful in distinguishing between different forms of the disorder. Comparing clustering results with and without the inclusion of these tests enabled a judgement as to whether they had a major impact on how patients were classified by the clustering algorithm.

### 2.5 Voxel-based morphometry

In addition to considering the linguistic and cognitive profiles of patients assigned to each cluster, we compared patterns of brain atrophy in each group. MRI scans for all patients were performed on a Siemens Trio 3T system (Siemens Medical Systems, Erlangen, Germany), with the exception of three patients who were not scanned due to contraindications. T1-weighted anatomical images were acquired using 3-dimensional magnetization-prepared rapid acquisition gradient echo and pre-processed using an automated pipeline (Acosta-Cabronero, Williams, Pereira, Pengas, & Nestor, 2008). All volumes were then spatially normalized, segmented, and smoothed using the unified segmentation model in SPM5. Total intracranial volumes were calculated using a validated method of summing grey matter, white matter, and CSF tissue classes (Pengas, Pereira, Williams, & Nestor, 2009) and the obtained values, along with age, were entered into the statistical models as nuisance covariates. The three groups of patients identified in the k-means analysis were each separately compared with the healthy control group to determine the main areas of atrophy associated with each group. Images were subjected to a statistical threshold of FDR *p* < 0.05.

## 3 Results

### 3.1 Principal components analysis

**Fig. 1:**
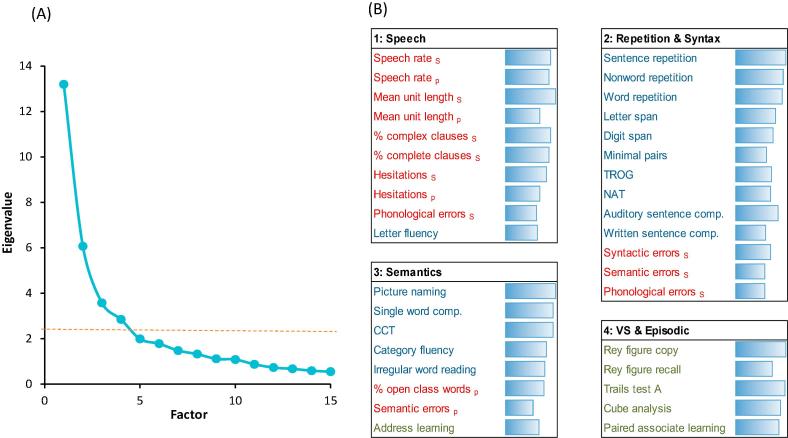
Results of principal components analysis. (A) Scree plot, indicating an elbow and marked reduction in eigenvalues after four factors. (B) Individual performance measures loading on each of the four factors. Measures with loadings >0.5 are listed. Connected speech markers are shown in red, neuropsychological language/semantic tests in blue and other neuropsychological tests in green. Bars indicate the strength of the loading of each measure. S and P sub-scripts denote speech markers derived from either semi-structured interviews or from picture description. TROG = Test of Reception of Grammar (Bishop, 1982), NAT = Northwestern Anagram Test (Weintraub et al., 2009), CCT = Camel & Cactus Test (Bozeat, Lambon Ralph, Patterson, Garrard, & Hodges, 2000).

As shown in Fig. 1A, there was a pronounced elbow in the scree plot after four factors, indicating that there was little explanatory benefit in extracting more than four factors from the data. The four-factor solution, which accounted for 61% of the variance, is presented in Fig. 1B and comprises four readily-interpretable factors. The first factor was comprised almost entirely of measures obtained from analysis of the patients’ connected speech samples. These measures index the fluency and complexity of speech production. The only neuropsychological test that loaded heavily on this factor was letter fluency, which also requires patient-initiated generation of speech.

Various tests of repetition, working memory, and syntactic processing loaded on the second factor. These included span for letters and digits, three receptive tests of grammatical processing which probed understanding of syntactically complex structures, and scores on the Northwestern Anagram Test (NAT), a sentence production task (Sajjadi et al., 2012, Weintraub et al., 2009). Speech error rates during semi-structured interviews also loaded on this factor, which may be indicative of syntactic deficits or working memory limitations leading to disconnected, error-prone speech.

The third factor was composed mainly of tests probing semantic knowledge, including picture naming, single-word comprehension and non-verbal semantic association. Semantic errors in picture description also loaded on this factor, as did the ratio of open to closed-class words. This may reflect the tendency for patients with semantic deficits to omit content words when describing events (Meteyard & Patterson, 2009). Score on the address recognition section of the ACE-R also loaded weakly on this factor. Although primarily a test of episodic memory, learning a name and address also depends on more general verbal semantic knowledge and thus might be expected to pattern with semantic tests. The final factor was composed entirely of non-linguistic neuropsychological tests, chiefly probing visuospatial ability and non-verbal episodic memory.

Each of the linguistic factors identified here – speech production, repetition and syntax, semantics – are key areas of difficulty for specific PPA subtypes. This confirms that our assessment probed relevant areas of impairment thought to distinguish between different forms of PPA. It is worth noting, however, that tests of repetition and syntax loaded on a single factor, despite these abilities dissociating in the criteria for lvPPA (i.e., impairment in repetition is a core diagnostic criterion for this variant but syntax is typically assumed to be spared). The results are in accord with a previous PCA performed on only a subset of the data presented here, which also produced factors corresponding to semantic ability, repetition and syntax, and quality of connected speech (Sajjadi et al., 2012). The main difference is that here we included non-linguistic neuropsychological tests, which loaded on a separate factor. We used patient scores on each of the PCA factors to interpret the results of the clustering analyses reported next.

### 3.2 K-means clustering of patients

**Fig. 2:**
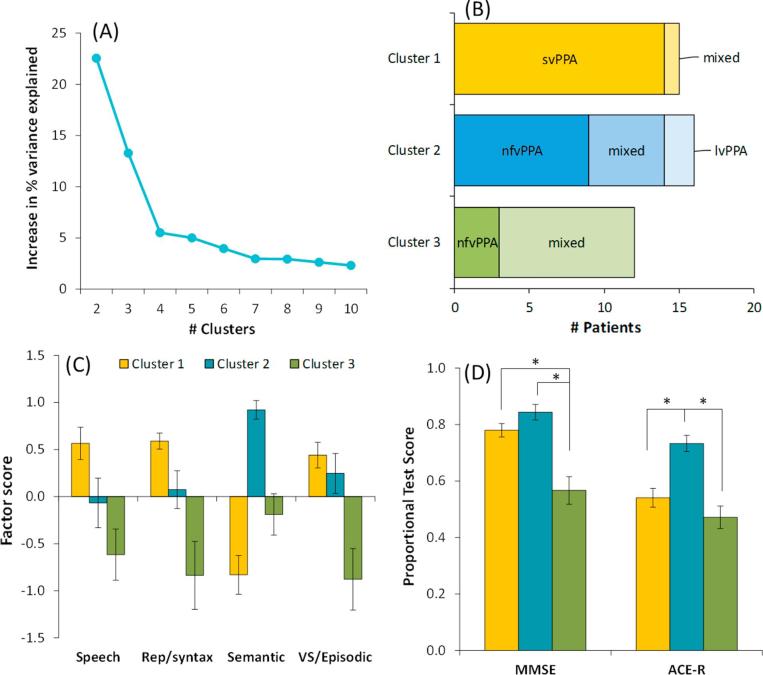
Results of k-means clustering including non-linguistic tests. (A) Increase in variance explained by the addition of each new cluster. There is considerable explanatory power in splitting the patients into two and then three clusters but little benefit derived from further sub-division. (B) Displays how patients with each clinical diagnosis were assigned in the three cluster solution. (C) Mean scores for patients in each cluster on the four factors identified by PCA. (D) Mean scores for patients in each cluster on the MMSE and ACE-R.

The k-means clustering on the full dataset varied the number of clusters between two and ten. As shown in Fig. 2A, dividing the patients into two and then three clusters each produced substantial increases in the variance explained by the clustering solution (∼23% and 13%). Further sub-division into four clusters yielded little additional explanatory power (∼5%). Our data therefore favour a tripartite division of PPA patients. The result was corroborated by the Bayesian model-based clustering algorithm (Fraley & Raftery, 2007), which statistically compared the strength of the evidence supporting solutions with varying numbers of clusters. This technique also indicated that a three-cluster solution was best supported by the data.

We next explored the characteristics of the three clusters of patients identified in the k-means analysis. Table 1 provides demographic information for patients in each group. There were no significant differences in age, educational level or disease duration. Fig. 2B displays membership of each cluster according to clinical diagnosis. Cluster 1 was composed almost entirely of svPPA patients, plus one patient with a diagnosis of mixed PPA (this individual did present with a clear semantic impairment but did not meet criteria for svPPA because he also had mild deficits in word and nonword repetition). The majority of the nfvPPA patients fell into Cluster 2, as did the two lvPPA patients and five of the mixed PPA cases. Cluster 3 mainly contained patients diagnosed with mixed PPA, as well as three nfvPPA cases. Therefore, although data-driven clustering supported a tripartite classification of patients with PPA, the three clusters do not correspond well with the diagnostic categories currently in use. svPPA patients were clearly separated from other forms of PPA but no such clear division was found for the other two subtypes.

**Table 1:** Demographic information for each cluster.

	Cluster 1	Cluster 2	Cluster 3	Controls	Omnibus ANOVA (*p* if < 0.05)
N	15	16	12	30	
Age, y	68.7 (61–79)	71.3 (63–79)	71.0 (53–83)	67.7 (51–80)	ns
Education, y	13.9 (10–19)	11.8 (9 −2 0)	11.7 (9 −1 8)	12.8 (10–20)	ns
Disease duration, y	4.2 (2.0–6.5)	3.0 (2.0–6.0)	3.3 (1.5–6.0)	–	ns

Mean values are shown, with range in parentheses. ns = not significant.

We explored the neuropsychological and spontaneous speech profiles of each cluster by calculating their mean scores on each of the four factors identified in the PCA. These results are shown in Fig. 2C (note that factor scores are scaled such that the mean for the whole cohort is zero). The profile for Cluster 1 was distinctive. These patients performed well in all domains except for Semantic, where they showed the greatest level of deficit. This is in line with the established profile of svPPA. In contrast to Cluster 1, Cluster 2 displayed good Semantic ability but somewhat lower scores on the other factors, particularly Speech and Repetition/Syntax. Impairment in these domains is central to the definitions of both nfvPPA and lvPPA. Cluster 3 patients showed low scores on all factors and were by far the poorest on the Speech, Repetition/Syntax and VS/Episodic factors. This profile of severe problems with speech production, repetition and syntax but also some weakness in semantic abilities does not correspond to any of the variants in the current consensus recommendations. Indeed, the majority of patients in this cluster were mixed cases who defied the accepted classifications. It is worth emphasising again that these cases were not simply at a more advanced stage of disease, at least as indexed by disease duration.

To summarise, the data-driven clustering approach neatly partitioned patients with selective semantic difficulties and divided the remaining patients into two groups. It is important to note, however, that the two other groups were differentiated primarily by severity of their impairment: the patients in Cluster 3 were more impaired in *all* domains than those in Cluster 2. This impression was confirmed by inspection of their scores on individual measures, shown in Supplementary Tables 1 and 2. Compared to Cluster 2, there were 17 measures on which Cluster 3 patients were significantly more impaired but no measures for which the reverse was true. This conclusion is also supported by scores on the MMSE and ACE-R (see Fig. 2D). One-way ANOVAs indicated a significant effect of cluster on each test (MMSE: *F*(2, 40) = 18.1, *p* < 0.001; ACE-R: *F*(2, 40) = 16.7, *p* < 0.001). Pairwise comparisons (conducted with a Bonferroni-corrected significance level of 0.0166) indicated that Cluster 3 patients scored more poorly than Cluster 2 individuals on both tests. Cluster 1 patients performed at a similar level to those in Cluster 2 on the MMSE but their scores on the ACE-R were poorer, being more comparable to Cluster 3. This reflects the fact that the ACE-R places greater emphasis on semantic abilities than does the MMSE.

### 3.3 Voxel-based morphometry

**Fig. 3:**
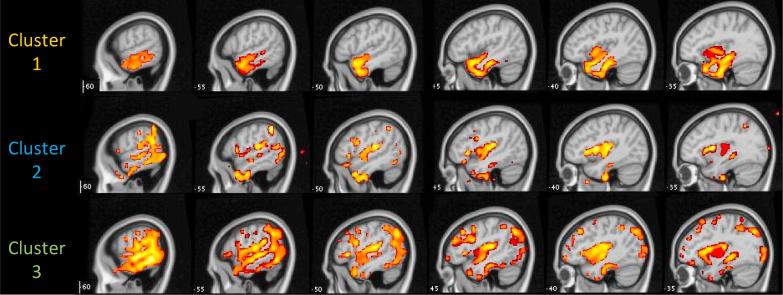
Voxel-based morphometry for each cluster of patients.

Areas of reduced grey matter density in each group are shown in Fig. 3. Cluster 1 patients displayed a distinctive pattern of bilateral anterior temporal lobe atrophy, more severe in the left hemisphere. This pattern is strongly associated with svPPA (Gorno-Tempini et al., 2004, Nestor et al., 2006). Atrophy in this group also extended into the left insula. Clusters 2 and 3 showed a wider distribution of damage. Both groups showed a strongly left-lateralised pattern, which encompassed posterior and anterior temporal cortex, inferior parietal cortex and the insula (bilaterally in Cluster 3). The spatial distribution of damage in the two groups was very similar, though damage was much more extensive in Cluster 3. This is consistent with the behavioural results, which indicated no qualitative differences between the two groups but more severe impairment across the board in Cluster 3. In Cluster 3, damage was also evident in the posterior hippocampus and posterior cingulate. These areas are among the first to be affected in typical AD (Nestor et al., 2006).

### 3.4 Cluster analyses excluding non-linguistic tests

**Fig. 4:**
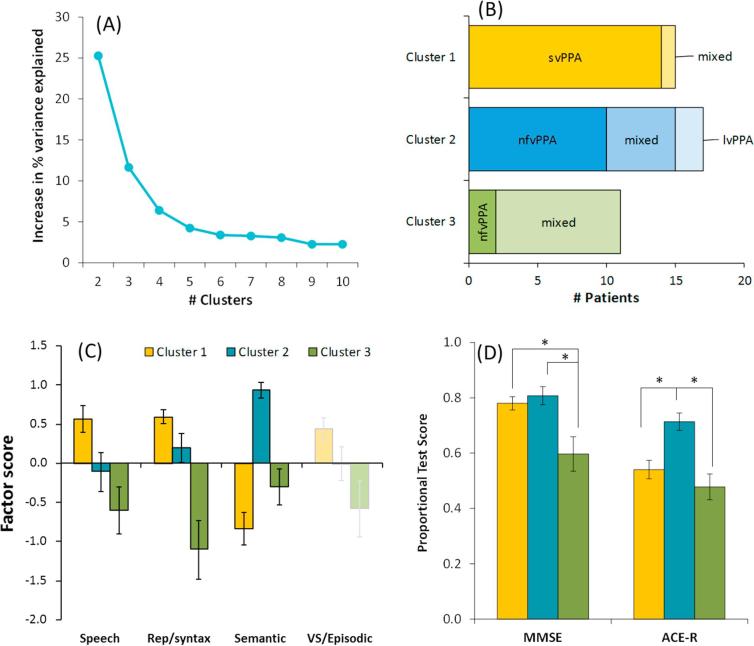
Results of k-means clustering excluding non-linguistic tests. (A) Increase in variance explained by the addition of each new cluster. Again, this indicates that division into three clusters provides a substantial gain in variance explained but there are diminishing returns from further sub-division. (B) Displays how patients with each clinical diagnosis were assigned in the three cluster solution. (C) Mean scores for patients in each cluster on the four factors identified by PCA. The VS/Episodic factor is faded to indicate that tests that load strongly on this factor were not included in the clustering computation. (D) Mean scores for patients in each cluster on the MMSE and ACE-R.

Repeating the k-means cluster analysis after excluding data from non-linguistic neuropsychological tests produced results that were very similar to those of the main analysis (see Fig. 4). Only three patients changed their cluster membership in the revised analysis. One nfvPPA patient moved from Cluster 2 to Cluster 3; one nfvPPA and one mixed PPA patient moved from Cluster 3 to Cluster 2. These results suggest that, although the three clusters differed significantly in their level of non-linguistic cognitive ability, scores on these tests did not have a major bearing on how the patients were partitioned by the clustering algorithm.

## 4 Discussion

The division of PPA patients into distinct variants of the disorder is an area of active debate. Some researchers have suggested that current diagnostic criteria (Gorno-Tempini et al., 2011) are too narrow to encompass the full range of symptom profiles present in patients (Harris et al., 2013, Mesulam and Weintraub, 2014, Sajjadi et al., 2012). Here, we used k-means clustering as a data-driven means of investigating clustering among 43 patients from the Cambridge longitudinal study of PPA (Sajjadi et al., 2012). The clustering algorithm we used was blind to diagnostic criteria and instead grouped patients together based on similarities in their linguistic and neuropsychological profiles. Although the optimum solution divided the patients into three groups, these groups did not map neatly on to the three proposed variants of the disorder. One group displayed a severe and selective semantic impairment, accompanied by pronounced atrophy to anterior temporal cortices, which corresponds closely to the criteria for svPPA. A second group exhibited good semantic performance and general cognition but were impaired in connected speech production, repetition and syntactic processing. This symptom profile is broadly consistent with at least parts of the proposed definitions for both nfvPPA and lvPPA. The final group manifested deficits in all domains tested, indicative of a mixed aphasic profile that matches none of proposed variants. Importantly, the key factor distinguishing Clusters 2 and 3 appeared to be overall severity, with Cluster 3 individuals showing greater impairments across the board. This conclusion was supported by examination of the extent of atrophy in each group: the two groups displayed similar spatial distribution of atrophy but the damage was more severe in Cluster 3. These results suggest that among non-svPPA patients, there are some individuals who present with a circumscribed language impairment, sparing semantic knowledge and general cognition, and others who demonstrate a wider range of more severe deficits. Finally, we found that inclusion of non-linguistic cognitive test scores had little bearing on how patients were divided into different clusters.

The clearest and least surprising finding in the study is that a cluster of PPA patients presented with a distinctive and homogeneous profile characterised by selective impairment in semantic processing and preserved function in other cognitive and linguistic domains. All but one of the patients in this cluster received a clinical diagnosis of svPPA. Measures loading on this factor included verbal and non-verbal tests of semantic knowledge, irregular word reading (which is thought to require support from the semantic system; Woollams, Lambon Ralph, Plaut, & Patterson, 2007) and some specific aspects of speech production in picture description, namely commission of semantic errors and reduced production of open-class words. All of these deficits are consistent with damage to a store of multimodal semantic knowledge (Lambon Ralph et al., 2010, Patterson et al., 2007). Previous studies of speech production in svPPA have indicated that loss of semantic knowledge leads to semantic paraphasias and the replacement of specific nouns and verbs with increasingly general terms and pronouns (Ash et al., 2006, Bird et al., 2000). In line with previous studies, we found that speech remains fluent and largely grammatically intact in these patients (Meteyard and Patterson, 2009, Sajjadi et al., 2012a). This cluster of patients displayed atrophy of the anterior temporal cortices, predominantly in the left hemisphere. This is a typical result for svPPA patients (Acosta-Cabronero et al., 2011, Rohrer et al., 2009, Rosen et al., 2002).

Distinctions among non-svPPA patients were less clear-cut. From a diagnostic perspective, one of the key goals in establishing diagnostic criteria is to aid in distinguishing between the two broad pathological categories that underpin non-semantic PPA: FTLD (comprising tau and TDP-43 pathology) and AD. Tau pathology is more common in patients diagnosed with nfvPPA (Josephs et al., 2006, Knibb et al., 2006), while markers of amyloid deposition and other indicators of AD pathology are more prevalent in those with lvPPA (Chare et al., 2014, Gil-Navarro et al., 2013, Leyton et al., 2011). These distinctions are not absolute, however, and some authors have argued that a lvPPA diagnosis is a poor guide to AD status (Harris et al., 2013, Rogalski et al., 2016, Sajjadi et al., 2014). To what extent has our data-driven analysis separated these two root causes? It is not possible to give a definitive answer to this question, as we do not have biomarker data or pathological disease confirmation for the majority of our patients. Analyses of cortical atrophy suggest, however, that it has not. Alzheimer-related PPA is typically associated with a posterior temporoparietal focus of atrophy while FTD degeneration has a fronto-insular focus (Gorno-Tempini et al., 2008, Hu et al., 2010, Kas et al., 2012, Rohrer et al., 2010). Our two non-svPPA clusters of patients displayed a mixed atrophy profile that encompassed both of these regions. The principal difference between groups was the degree of atrophy rather than its spatial distribution. This suggests that the two groups are likely to contain cases of both forms of pathology and therefore that the *overall* linguistic and cognitive profiles of AD and non-AD cases of (non-semantic) PPA are not markedly different. This finding offers data-driven evidence to corroborate the clinical impression that nfvPPA and lvPPA can be very hard to differentiate (Leyton et al., 2011). Of course, more specific features may be of greater diagnostic value. It has recently been suggested that phonological error rates show a strong association with levels of amyloid deposition (Leyton et al., 2014, Mesulam et al., 2012). These error rates did not differ between our two clusters, however.

Finally, our study differs from previous data-driven investigations of PPA patients in that we took into account performance on a range of non-linguistic neuropsychological tests. The svPPA cluster performed reasonably well in these domains, as did the less severe non-svPPA group (Cluster 2). Cluster 3, however, showed deficits extending beyond the language domain, affecting visuospatial function and episodic memory. This was corroborated by poorer scores on the MMSE and ACE-R. Impairment to multiple cognitive domains has previously been reported in lvPPA patients as their disease progresses, in contrast to the circumscribed deficits observed in svPPA (Leyton et al., 2013). None of the patients in Cluster 3 were diagnosed with lvPPA, however. Instead, the majority were unclassifiable mixed PPA patients who had severe and broad language deficits and widespread cortical atrophy. Thus, our results suggest that impairments in PPA extend beyond language in many cases but that such deficits are not specific to patients who meet the criteria for lvPPA. Importantly, consideration of non-linguistic test scores had little effect on the number or composition of the data-driven patient clusters, with similar results when these tests were removed from the analysis (see Fig. 4). Nevertheless, our results suggest that while extra-linguistic cognitive impairments go hand in hand with more severe language impairments, these do not necessarily add significant value in characterising different forms of the disorder. We note, however, that our non-linguistic tests probed only visuospatial ability and episodic memory. It is possible that the use of a wider range of tests, including tests of executive function, would have greater utility.

## Statement of significance

This study addresses ongoing debate concerning how patients with PPA should be grouped into different variants of the disorder. This is important as different PPA presentations are associated with different forms of pathology. Results suggest that the current classification system does not adequately account for the full range of presentations.
