# Supplementary material for: Data-driven classification of patients with primary progressive aphasia
Source: Brain Lang. 2017 Nov;174:86–93. doi: 10.1016/j.bandl.2017.08.001 (PMC5626563; doi:10.1016/j.bandl.2017.08.001)
Supplement: Supplementary Tables 1 and 2 [file mmc1.docx]

Supplementary Table 1: Mean scores for each group on individual neuropsychological tests

| Test (maximum) | Cluster 1 | Cluster 2 | Cluster 3 | Controls | Omnibus ANOVA (*p* if <0.05) |
| --- | --- | --- | --- | --- | --- |
| *General* |  |  |  |  |  |
| ACE-R address recognition (5) | 2.9*^a^ (0-5) | 4.5^ac^ (2-5) | 2.3*^c^ (0-5) | 4.9 (3-5) | <.001 |
| VOSP cube analysis (10) | 9.9^ab^ (9-10) | 8.5^ac^ (5-10) | 5.6^bc^ (2-10) | NA |  |
| Rey figure copy (36) | 33.3^ab^ (29-36) | 26.3^a^ (0.5-36) | 17.6^b^ (2.5-33) | NA |  |
| Rey figure recall (36) | 10.8 (0-25) | 10.7 (0-23) | 4.8 (0-14.5) | NA |  |
| Trails A (sec) | 51.3^b^ (21-98) | 79.6 (47-185) | 159.0^b^ (48-394) | NA |  |
| Paired associates learning (errors) | 13.6^b^ (2-28) | 16.3^c^ (0-49) | 50.1^bc^ (2-78) | NA |  |
| Letter fluency (words/min) | 5.9* (1-11) | 5.6* (1-11) | 4.3* (1-8) | 14.4 (4-21) | <.001 |
| *Repetition/Short-term Memory* |  |  |  |  |  |
| Forward digit score (12) | 8.1^ab^ (4-12) | 5.1*^ac^ (1-8) | 2.9*^bc^ (1-5) | 7.8 (5-11) | <.001 |
| Backward digit score (12) | 6.6^ab^ (4-11) | 4.2*^a^ (3-6) | 2.9*^b^ (1-7) | 6.3 (3-10) | <.001 |
| Serial recall of letter lists (24) | 13.4^ab^ (9-21) | 9.1*^ac^ (5-12) | 5.9*^bc^ (1-9) | 14.0 (9-21) | <.001 |
| Minimal pairs discrimination (48) | 41.9^ab^ (37-46) | 37.8*^a^ (26-43) | 32.9*^b^ (23-43) | 41.4 (32-47) | <.001 |
| Single word repetition (22) | 20.6 ^ab^ (17-22) | 17.8*^ac^ (13-21) | 14.3*^bc^ (5-21) | 21.2 (18-22) | <.001 |
| Nonword repetition (12) | 10.3^ab^ (7-12) | 7.2*^a^ (0-11) | 5.1*^b^ (0-10) | 10.7 (8-12) | <.001 |
| Sentence repetition (24) | 21.9*^ab^ (18-24) | 16.9*^ac^ (6-23) | 9.3*^bc^ (0.5-19) | 23.7 (21.5-24) | <.001 |
| *Syntax* |  |  |  |  |  |
| TROG (28) | 26.9^b^ (25-28) | 25.6^c^ (16-28) | 17.3*^bc^ (8-26) | 27.8 (27-28) | <.001 |
| Auditory sentence comprehension (24) | 18.5*^b^ (8-23) | 14.8*^c^ (6-23) | 7.3*^bc^ (0-14) | 22.7 (18-24) | <.001 |
| Written sentence comprehension (24) | 19.3*^b^ (12-24) | 19.1*^c^ (13-24) | 11.8*^bc^ (7-18) | 23.0 (18-24) | <.001 |
| MAST (30) | 13.8*^b^ (0-29) | 15.3*^c^ (0-27) | 4.6*^bc^ (0-16) | 24.9 (13-30) | <.001 |
| NAT (10) | 7.2^b^ (1-10) | 6.0*^c^ (0-9) | 2.3*^bc^ (0-5) | 9.1 (0-10) | <.001 |
| *Semantics* |  |  |  |  |  |
| Single word comprehension (10) | 4.7*^ab^ (1-9) | 9.4^ac^ (6-10) | 7.3*^bc^ (4-10) | 9.7 (9-10) | <.001 |
| Camel & Cactus Test (64) | 31.1*^a^ (0-51) | 52.7*^ac^ (43-61) | 40.9*^c^ (12-60) | 58.8 (53-63) | <.001 |
| Irregular word reading (5) | 2.6*^a^ (0-5) | 4.8^a^ (1-5) | 3.3* (1-5) | 4.9 (4-5) | <.001 |
| Picture naming (64) | 22.3*^ab^ (4-51) | 59.6^ac^ (46-64) | 46.3*^bc^ (14-63) | 62.3 (57-64) | <.001 |
| Category fluency (words/min) | 4.3*^a^ (0-11) | 10.1*^a^ (2-16) | 6.9* (2-16) | 19.7 (13-31) | <.001 |

* = cluster differs significantly different from controls. a = significant difference between Clusters 1 and 2. b = significant difference between Clusters 1 and 3. c = significant difference between Clusters 2 and 3. NA indicates control data not available. A Bonferroni-corrected significance level of *p*< 0.0083 was adopted for all pairwise comparisons.

Supplementary Table 2: Mean scores for each group on individual connected speech markers

| Marker | Cluster 1 | Cluster 2 | Cluster 3 | Controls | Omnibus ANOVA (*p* if <0.05) |
| --- | --- | --- | --- | --- | --- |
| *Semi-Structured Interviews* |  |  |  |  |  |
| Mean unit length | 7.8*^b^ (5.2-10.1) | 6.2* (3.5-11.3) | 4.6*^b^ (2.7-7.3) | 9.7 (6.4-14.8) | <.001 |
| % complete clauses | .82^b^ (.64-.94) | .73*^c^ (.54-.94) | .54*^bc^ (.32-.84) | .89 (.63-1) | <.001 |
| % complex clauses | .28* (.05-.57) | .18* (0-.58) | .11* (0-.42) | .48 (.11-1) | <.001 |
| Speech rate, words per minute | 109.7*^ab^ (59-163) | 73.6*^a^ (21-113) | 62.4*^b^ (24-90) | 140.9 (90-190) | <.001 |
| Hesitation rate | .02^ab^ (.01-.06) | .08*^a^ (.02-.23) | .12*^b^ (.05-.24) | .03 (0-.09) | <.001 |
| Semantic error rate | .07* (0-.21) | .02 (0-.17) | .11* (0-.47) | .00 (0-.08) | <.001 |
| Phonological error rate | .01^ab^ (0-.02) | .02*^a^ (0-.07) | .04*^b^ (.01-.11) | .00 (0-.01) | <.001 |
| Syntactic error rate | .05 (0-.20) | .09* (0-.26) | .10* (0-.23) | .03 (0-.12) | <.001 |
| % open class words | .41 (.30-.58) | .39* (.28-.55) | .36* (.25-.48) | .45 (.33-.64) | .004 |
| *Picture Descriptions* |  |  |  |  | <.001 |
| Mean unit length | 8.3*^b^ (5.3-13.0) | 8.0* (5.5-13.0) | 5.8*^b^ (3.1-8.0) | 12.9 (8.0-26.7) | <.001 |
| % complete clauses | .85* (.57-1) | .81* (.50-1) | .71* (.43-.88) | .95 (.75-1) | <.001 |
| % complex clauses | .28* (0-1) | .23* (0-.67) | .12* (0-.33) | .56 (.07-1.33) | <.001 |
| Speech rate, words per minute | 109.3*^ab^ (46-157) | 67.5*^a^ (26-116) | 59.9*^b^ (14-114) | 139.5 (90-198) | <.001 |
| Hesitation rate | .01^ab^ (0-.05) | .07*^a^ (0-.24) | .10*^b^ (0-.06) | .02 (0-.06) | <.001 |
| Semantic error rate | .27* (0-1) | .09* (0-.33) | .14* (0-.57) | .01 (0-.06) | <.001 |
| Phonological error rate | .01^a^ (0-.04) | .03*^a^ (0-.09) | .02* (0-.09) | .00 (0-.01) | <.001 |
| Syntactic error rate | .03 (0-.13) | .12 (0-.67) | .09 (0-.26) | .04 (0-.16) | .020 |
| % open class words | .35*^a^ (.26-.43) | .41^ac^ (.36-.48) | .35*^c^ (.25-.45) | .40 (.32-.47) | <.001 |

* = cluster differs significantly different from controls. a = significant difference between Clusters 1 and 2. b = significant difference between Clusters 1 and 3. c = significant difference between Clusters 2 and 3. A Bonferroni-corrected significance level of *p*< 0.0083 was adopted for all pairwise comparisons.
